# Antioxidant and Anti-Inflammatory Effects of *Origanum majorana* L. Methanolic Extract on Bile Duct Ligation in Male Rats

**DOI:** 10.1155/2021/9927196

**Published:** 2021-05-07

**Authors:** Izadpanah Gheitasi, Nikta Motaghi, Hossein Sadeghi, Heibatollah Sadeghi, Zahra Moslemi, Mahdieh Eftekhari, Nasrin Shakerinasab, Amir Hossein Doustimotlagh

**Affiliations:** ^1^Medicinal Plants Research Center, Yasuj University of Medical Sciences, Yasuj, Iran; ^2^Student Research Committee, Yasuj University of Medical Sciences, Yasuj, Iran; ^3^Department of Pharmacognosy and Pharmaceutical Biotechnology, Faculty of Pharmacy, Kermanshah University of Medical Sciences, Kermanshah, Iran

## Abstract

**Introduction:**

Cholestasis is caused by malfunction of the hepatobiliary system. This disorder is the result of the accumulation of bile fatty acids and other toxins in the liver. The aim of the current study was to investigate the antioxidative and hepatoprotective effects of methanolic extract of Origanum *majorana* L. (OM) on hepatic disorder and tissue damage induced by bile duct ligation (BDL) in rats. *Materials and methods*. Twenty-eight male Wistar rats were randomly divided into 4 groups including sham control group received vehicle (SC-V), bile duct ligation received vehicle (BDL-V), bile duct ligation group received OM extract (BDL + OM), and sham control group received OM extract (SC + OM). One day after surgery, the animals received vehicle or methanolic extract of OM 300 mg/kg/day for 7 consecutive days by oral gavage. Finally, the animals were anesthetized and the blood samples were collected from each animal. After sacrificing of animals, liver tissue from each rat was removed and divided into three parts: one part was used for preparing of homogenized tissue, one part was fixed in 10% neutral formalin for histopathology examination, and the third part was kept in liquid nitrogen for gene expression analysis. Biomarkers of oxidative stress in the liver tissue and serum, as well as histopathological changes of the liver, were assessed. Also, the gene expression of IL-1, TNF-*α*, TGF-*β*, and *α*-SMA has been measured.

**Results:**

The results showed that BDL-V significantly increased the activity of ALT, AST, ALP, and total bilirubin compared to the SC-V group. The oxidative stress markers such as MDA and FRAP significantly increased due to BDL, while the CAT activity reduced in the BDL-V group compared to SC-V group. Oral treatment with OM reduced ALT and AST activity, although it was not statistically significant. OM treatment considerably increased the activity of CAT compared to BDL group. BDL-V induced a significant histological change in the liver, while treatment with OM at a dose of 300 mg/kg showed a minor effect on histopathological changes. In addition, the mRNA of IL-1, TNF-*α*, TGF-*β*, and *α*-SMA significantly increased in the BDL-V group, while treatment with OM only significantly reduced TGF-*β* in comparison with BDL-V rats.

**Conclusions:**

The results of the present study showed that oral administration of OM extract had a moderate protective effect on cholestasis due to BDL. Indeed, more studies with different doses of extract are needed to confirm this finding.

## 1. Introduction

Liver is one of the main organs of the body that is involved in providing energy, excretion of harmful substances and drugs, and maintaining and regulating homeostasis [1, 2]. Hepatic cholestasis is due to defect in the production, secretion, and flow of bile which causes the accumulation of bile acids in the liver. Bile acids accumulated in pathological concentrations cause the inflammation of liver tissue, destruction of hepatocytes, and the production of reactive oxygen species (ROS) and the development of oxidative stress [1, 3]. Intrahepatic cholestasis can be inherited or acquired, such as drug-induced liver injury, pregnancy, hepatitis, and primary biliary cirrhosis. In untreated cases, hepatic cholestasis can cause itchy skin, hyperlipidemia, and jaundice, which progresses to liver cirrhosis, fibrosis, and liver failure. Ursodeoxycholic acid is currently the only Food and Drug Administration (FDA) approved drug for the treatment of cholestasis that has not seen improvement in cholestasis symptoms in some patients after taking this drug [4]. Despite liver disorders being a global problem with high mortality and morbidity, there is no effective treatment to control their progression. New drugs for the treatment of chronic liver disorders have also been found to have harmful effects [5]. The phenomenon of oxidative stress is due to the overproduction of ROS, which leads to an imbalance of antioxidant and pro-oxidative processes [6]. ROS contains a large group of molecules such as peroxyl radical, superoxide radical, nitric oxide, hypochlorous acid, hydroxyl radical, and hydrogen peroxide [7]. Antioxidants can produce harmless products by reacting with free radicals. Catalase (CAT), superoxide dismutase (SOD), glutathione reduced (GSH), and glutathione peroxidase (GPX) are a group of endogenous antioxidants [8]. Marjoram plant with the scientific name of *Origanum majorana* L (OM) belongs to the Lamiaceae family and grows mostly in America, Africa, Asia, Europe, and Arabian Peninsula [9]. Marjoram leaves are used in cases such as asthma, nervous problems, insomnia, and digestive disorders [10]. Pharmacological research has shown that OM has antifungal, antioxidant, anti-spasmodic, analgesic, antibacterial, antimutagenic, antitumoral, antithrombin, and antihyperglycaemic effects [11]. Research also has shown that ursolic acid of OM and its essential oil, especially thymol and carvacrol, have antiviral and bactericidal, antiseptic, and antifungal properties [12]. Ursolic acid and rosmarinic acid, as the isolated compounds from OM, also have liver protective and fat reducing properties [1, 13]. Bile duct ligation is an in vitro model of hepatic cholestasis induction that causes secondary biliary cirrhosis and oxidative stress, which increases liver function markers in animal models [14, 15]. Studies in CCl_4_-intoxicated rats have shown that methanolic extract of OM significantly reduces aspartate aminotransferase (AST), alanine aminotransferase (ALT), alkaline phosphatase (ALP), and its protective effect on the liver, possibly due to its inhibitory effect on cytochrome P450 and thus preventing the formation of free radicals [16]. The present study was designed to examine the effect of OM methanolic extract against oxidative damage, oxidative stress markers, and antioxidant status in BDL-induced hepatic injury in rats.

## 2. Material and Methods

### 2.1. Chemicals

Ethylenediaminetetraacetic acid (EDTA), pentobarbital, thiobarbituric acid (TBA), and 5,5′-dithiols-(2-nitrobenzoic acid) (DTNB) were purchased from Sigma Chemical Co (St Louis, MO, USA). Trichloroacetic acid (TCA), 2,4-dinitrophenylhydrazine (DNPH), and formaldehyde were obtained from Merck (Germany).

### 2.2. Preparation of *Origanum majorana* Methanolic Extract

In the summer of 2020, fresh aerial parts of *Origanum majorana* were collected from the foothills of Dena Mountain and then identified by the botanist (herbarium No: MPISB158).

*Origanum majorana* aerial parts were cleaned, then placed in room air, kept away from direct light for several days to dry, and then powdered. 100 grams of dried powder plant was soaked in 1000 ml of 70% methanol (methanol: water 70 : 30) and was placed at 37°C for 48 hours. The resulting mixture was centrifuged and filtered through Whatman filter paper NO. 1; after that, it was concentrated as much as possible under a vacuum rotary apparatus. Then, the extract was then dried in an incubator at 40°C and stored in a freezer at −20°C for the next experiments [17].

### 2.3. Animals and Experimental Design

In this study, 28 male Wister rats (3 months old, 200 to 250 g) were purchased from Shahrekord University of Medical Sciences. The rats were kept in animal laboratory of Yasuj University of Medical Science under the 12-hour light/dark cycle condition, and they had free access to tap water and food (Ethical code; IR.YUMS.REC.1398.158).

Rats were randomly divided into 4 groups of 7 animals each as follows: group 1: sham control + vehicle (SC-V), in which surgery was performed but the bile duct was not closed; group 2: cholestatic group + vehicle (BDL-V), in which surgery was performed with bile duct closure; group 3: SC + OM extract; group 4: BDL + OM. BDL was performed under antiseptic conditions and the animals received daily the methanolic extract of OM orally 300 mg/kg for 7 consecutive days. To do bile duct ligation on experimental groups, after induction of general anesthesia with ketamine-HCL 50 mg/kg and xylazine-HCL 10 mg/kg, the common bile duct of the animal was tied on two points and cut in half [18]. The day after surgery, groups 1 and 2 received normal saline and groups 3 and 4 received 300 mg/kg OM for 7 consecutive days. One week later, animals were anesthetized with ether, and the blood was collected by puncturing the heart for biochemical tests. Liver tissue was removed after collecting the blood. Three parts of the liver tissue were allocated; one of them was kept for gene expression analysis in liquid nitrogen, one of them was stored for histological examination in 10 percent formalin, and the third section was homogenized (10%, w/v) in PBS (10 mmol/L, pH 7.4).

### 2.4. Biochemical Analysis

Plasma was separated by centrifugation at 3,000 rpm at room temperature for 15 min. The activity of liver enzymes including ALT, AST, and ALP, and the level of total protein and total bilirubin were measured colorimetry using Pars Azmoon kit.

### 2.5. Measurement of Hydroxyproline Content

The level of tissue hydroxyproline was determined based on previous study [1]. 0.1 g of liver tissue was hydrolyzed in 6N HCl for 18h, Chloramine-T reagent and citrate buffer were added; then, fresh Ehrlich's reagent (15 g of p-dimethyl amino benzaldehyde in n-propanol/perchloric acid; 2 : 1 v/v) was added and placed in a warm water bath (60°C) for 15 min. Finally, the optical density was determined spectrophotometrically at 550 nm.

### 2.6. Oxidative Stress Markers

#### 2.6.1. Measurement of Ferric Reducing Antioxidant Power (FRAP)

In this method, the ability of plasma and homogenized tissue to reduce ferric ions (Fe^3+^) was measured. By reducing the ferric ions and converting them to Fe^2+^ at an acidic pH in the presence of TPTZ, the Fe-TPTZ complex was formed, which was blue. The resulting color intensity was measured spectrophotometrically at wavelength of 593 nm [19].

#### 2.6.2. Measurement of Malondialdehyde

The concentration of malondialdehyde (MDA) in the homogenized tissue and plasma was measured after reaction with thiobarbituric acid. The optical absorption of the red product was measured at 535 nm [20].

#### 2.6.3. Measurement of Total Thiol

First, 790 *μ*l of methanol and 150 *μ*l of Tris base-EDTA buffer were added to the test tube and then 50 *μ*l of homogenized tissue was added to it. After complete mixing, 50 *μ*l of DNTB reagent was added and vortexed again. The tubes were incubated for 15 minutes at room temperature and their OD was read at 412 nm [20].

### 2.7. Determination of Antioxidant Enzymes

#### 2.7.1. Measurement of Superoxide Dismutase Activity

The SOD activity was measured based on its ability to inhibit the oxidation of NADPH. According to this method, the superoxide radical was formed during a chemical reaction of molecular oxygen in the presence of EDTA, manganese chloride, and mercaptoethanol (oxidation of NADPH depends on the availability of the superoxide anion). When SOD was added to the measurement mixture, nucleotide oxidation was prevented. Nucleotide oxidation changes at 340 nm at physiological pH were measured spectrophotometrically [21].

#### 2.7.2. Measurement of Catalase Activity

Catalase activity was measured by Hugo Aebi method. In this method, the decomposition rate of H_2_O_2_ substrate at 420 nm was measured with a spectrophotometer [22].

### 2.8. Gene Expression of TNF-*α*, IL-1, TGF-*β*, and *α*-SMA

Real-time PCR calculated hepatic TNF-alpha, IL-1, *α*-SMA, and TGF-*β* mRNA. Total RNA was isolated from hepatic tissues using RNX Plus (Sinaclon, Tehran, Iran) and cDNA was generated on the basis of the manufacturer's technique from total RNA by cDNA Synthesis kit (Sinaclon, Tehran, Iran). RT-PCR was conducted using the instrument Rotor Gene 3000 (Bio-Rad, USA). The mRNA values of TNF-*α*, IL-1, *α*-SMA, and TGF-*β* were standardized to GAPDH. The PCR was completed in 40 cycles: TNF-*α* and IL-1 at 95°C for 15 seconds, 58°C for 30 seconds, and 72°C for 30 seconds, *α*-SMA at 95°C for 15 seconds, 63°C for 30 seconds, and 63°C for 30 seconds, and TGF-*β* at 95°C for 15 seconds, 55°C for 30 seconds, 72°C for 30 seconds. Data of TNF-*α*, IL-1, *α*-SMA, and TGF-*β* were calculated relative to GAPDH using the 2^–ΔCt^ method.

### 2.9. Histological Examinations

For evaluation of alterations at the histological level, liver tissue portions of the right lobe were stored in 10 percent formalin. It was cleared with xylene after dehydration in the graded alcohol series. The tissues were embedded in paraffin sectioned and stained with haematoxylin and eosin reagent. The sections under a light microscope have been studied.

### 2.10. HPLC Analysis

An adept series gradient high-performance liquid chromatography (HPLC) (Knauer 2500 basic model) system was utilized for chromatographic analysis. The HPLC was equipped with a valve for the injection of sample with a 20 *µ*L sample loop, a detector of UV/Vis, a quaternary pump, a per column (particle size of 5 *µ*m, Eurospher 100-5 Cis), and in-line vacuum solvent degasser. The mobile phase consisted of water-acetonitrile with v/v% of 30 : 70 a flow rate 1.1 ml min^−1^. A wavelength of 220 nm was applied for the system, and the processes of separation and detection were carried out at ambient temperature.

### 2.11. Statistical Analysis

The obtained data were statistically analyzed using SPSS software (Version 16). First, the normality of data was determined by Kolmogorov-Smirnov test; then one-way analysis of variance was used for parametric data with Tukey post hoc test. The data were reported as mean ± SEM. *P* < 0.05 was reported as significant for all data.

## 3. Results

### 3.1. Biochemical Parameters

**Figure 1:**
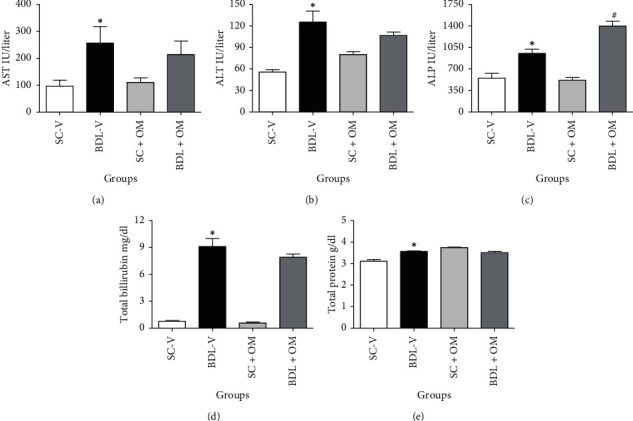
Effect of OM extract on biochemical markers. Each value represents the mean ± SEM. ^*∗*^Significantly different from SC-V, *P* value ≤ 0.05. ^#^Significantly different from BDL-V, *P* value ≤ 0.05. AST, aspartate aminotransferase (a); ALT, alanine aminotransferase (b); ALP, alkaline phosphatase (c); total bilirubin (d); total protein (e); SC-V, sham control + vehicle; BDL-V, bile duct-ligation + vehicle; OM, *Origanum majorana*.

As presented in Figure 1, the level of AST, ALT, ALP, total bilirubin, and total protein increased significantly in the BDL group compared to SC-V group (*P* < 0.05). Treatment of OM extract at a dose of 300 mg/kg in the cholestatic group slightly decreased the content of AST, ALT, total bilirubin, and total protein as compared to the BDL-V group.

### 3.2. Hydroxyproline Content

**Figure 2:**
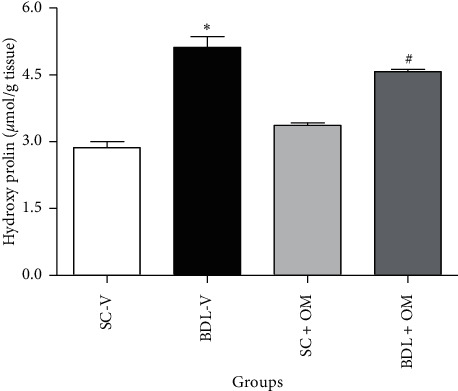
Effect of OM on hydroxyproline level. Each value represents the mean ± SEM. ^*∗*^Significantly different from SC-V, *P* value ≤ 0.05. ^#^Significantly different from BDL-V, *P* value ≤ 0.05. SC-V, sham control + vehicle; BDL-V, bile duct-ligation + vehicle; OM, *Origanum majorana*.

As presented in Figure 2, the level of hepatic hydroxyproline increased in BDL-V rats compared to SC-V group (*P* < 0.05). Treatment of animals with OM significantly decreased hydroxyproline (*P* < 0.05).

### 3.3. Plasma Oxidative Stress Markers

**Figure 3:**
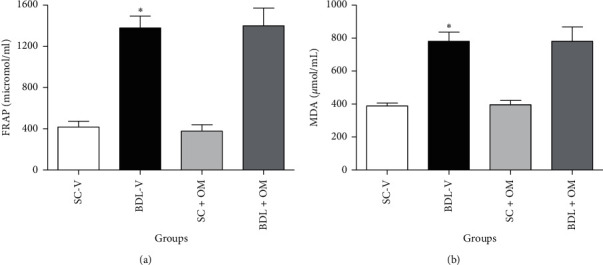
Effect of OM on FRAP (a) and MDA (b) plasma levels. Each value represents the mean ± SEM. ^*∗*^Significantly different from SC, *P* value ≤ 0.05. FRAP, ferric reducing antioxidant power; MDA, malondialdehyde; SC-V, sham control + vehicle; BDL-V, bile duct-ligation + vehicle; OM, *Origanum majorana*.

Figure 3 shows that FRAP and MDA levels in the BDL-V group increased significantly compared to the SC-V group (*P* < 0.05), but our analysis showed that OM had no significant effect on plasma oxidative stress markers in the BDL groups.

### 3.4. Liver Oxidative Stress Markers

**Figure 4:**
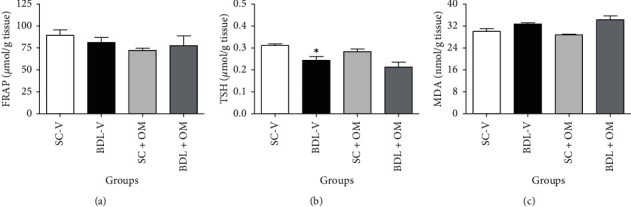
Effect of OM on the tissue oxidative stress markers. FRAP, ferric reducing activity of plasma. (a) TSH, total thiol. (b) MDA, malondialdehyde. (c) Each value represents the mean ± SEM. ^*∗*^Significantly different from SC-V, *P* value ≤ 0.05. SC-V, sham control + vehicle; BDL-V, bile duct-ligation + vehicle. OM, *Origanum majorana*.

As illustrated in Figure 4 FRAP and MDA levels did not change in the BDL-V group compared to the SC-V group. TSH level in the BDL-V group significantly decreased as compared to the SC-V group (*P* < 0.05). Our finding also showed that OM at a dose of 300 mg/kg had no significant effect on the FRAP, TSH, and MDA levels as compared to the BDL-V group.

### 3.5. Antioxidants Enzymes

**Figure 5:**
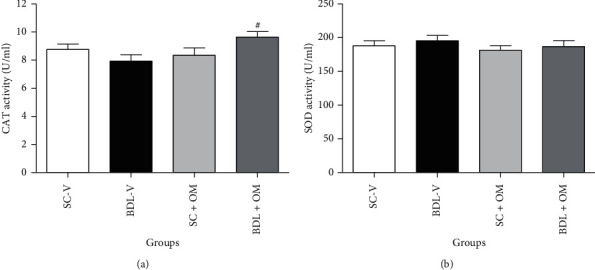
Effect of OM on CAT (a), SOD (b) activity in the liver tissue. Each value represents the mean ± SEM. SC-V, sham control + vehicle; BDL-V, bile duct-ligation + vehicle; OM, *Origanum majorana*; CAT, catalase; SOD, superoxide dismutase. ^#^Significantly different from BDL-V group, *P* ≤ 0.05.

As shown in Figure 5, CAT and SOD activity did not alter in the BDL-V group compared to the SC-V group. Administration of OM at a dose of 300 mg/kg caused a significant increase in CAT activity in the BDL group in contrast to the BDL-V group but it had no significant effect on SOD activity.

### 3.6. Histopathological Examination

**Figure 6:**
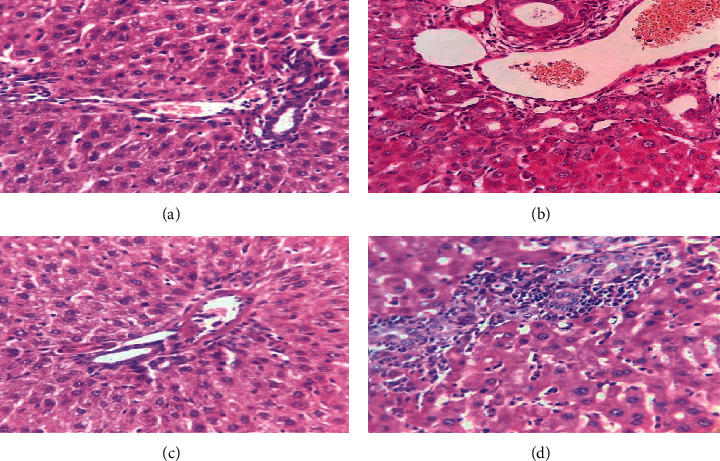
Liver section of the SC-V group (a), BDL-V group (b), SC + OM group (c), and BDL + OM group (d).

**Figure 7:**
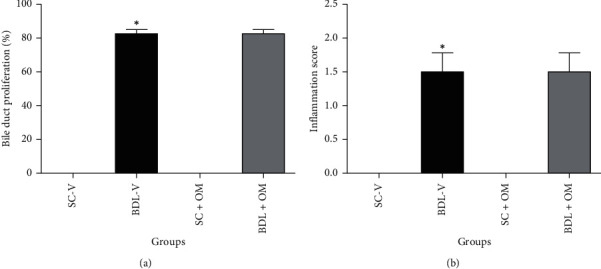
Effect of OM on bile duct proliferation (a), and inflammation of hepatic tissue (b). SC-V, sham control + vehicle; BDL-V, bile duct-ligation + vehicle and OM, *Origanum majorana*. ^*∗*^Significantly different from SC-V groups, *P* ≤ 0.05. ^#^Significantly different from BDL-V group, *P* ≤ 0.05.

Histological studies of SC-V and SC + OM groups revealed that the liver had a normal hepatic lobule structure (Figures 6(a) and 6(c)). Hepatic sections of BDL-V rats showed activation of hepatocytes and areas of hepatic necrosis (Figure 6(b)). Also, the liver of BDL rats treated with OM did not show significant improvement in the histopathological changes (Figure 6(d)). Examination of liver tissue showed that bile duct proliferation and inflammation increased significantly (80%) in the BDL group compared to the SC-V rats (*P* < 0.05), and OM had no effect on inflammation score of hepatic tissue (Figure 7).

### 3.7. Gene Expression of TNF-*α*, IL-1, *α*-SMA, and TGF-*β*

**Figure 8:**
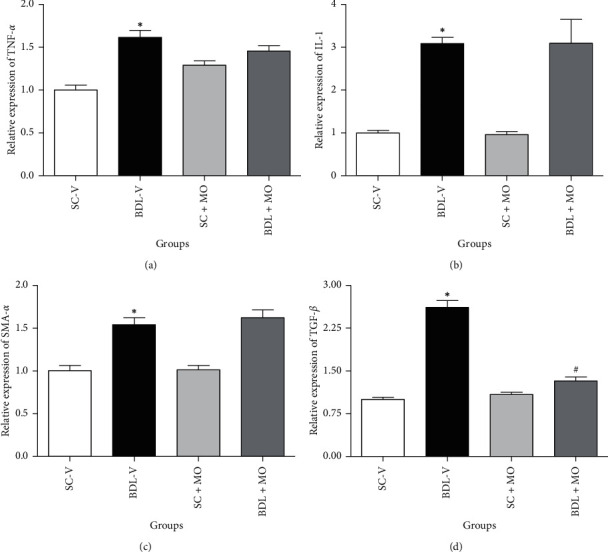
The effect of OM on the mRNA levels of TNF-*α*, IL-1, TGF-*β*, and *α*-SMA in BDL-induced cholestatic rats. SC-V, sham control + vehicle; BDL-V, bile duct-ligation + vehicle and OM, *Origanum majorana*. ^*∗*^Significantly different from SC-V groups, *P* ≤ 0.05. ^#^Significantly different from BDL-V group, *P* ≤ 0.05.

The mRNA levels of TNF-*α*, IL-1, *α*-SMA, and TGF-*β* significantly elevated in the liver tissue of BDL-V group in contrast to SC-V rats (*P* ≤ 0.05). Our results indicated that OM at a dose of 300 mg/kg had no significant effect on expression of TNF-*α*, IL-1, and *α*-SMA levels against the BDL-V rats, but significantly reduced the relative expression of TGF-*β* against the BDL-V rats (*P* ≤ 0.05) (Figure 8).

### 3.8. HPLC Analysis

**Figure 9:**
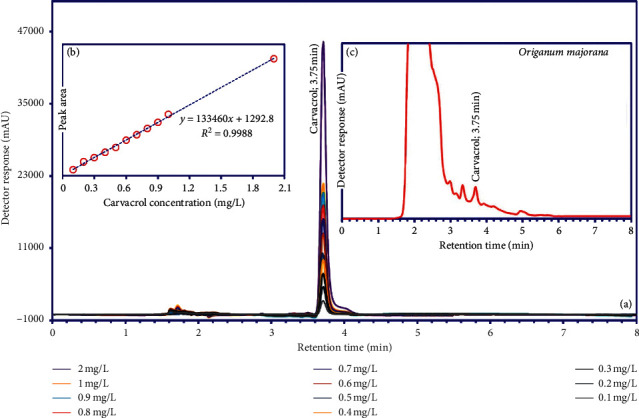
HPLC analysis of carvacrol in the methanolic extraction of *Origanum majorana*.

Typical analysis of the standard carvacrol in aqueous solution, related calibration curve, and methanolic extraction of OM collected at 8 min are shown in Figure 9(a). The calibration curve (Figure 9(b)) was prepared with carvacrol and was found to be linear (*R*^2^ = 0.9988) in the concentration range (0.10-2 mg L^−1^) used. Based on the HPLC analysis, the concentration of carvacrol on the extract of OM was 33.65 ± 1.87 mg g^−1^ (Figure 9(c)).

## 4. Discussion

Hepatic cholestasis is one of the causes of liver cirrhosis and fibrosis. Nowadays, researchers are trying to understand the mechanisms associated with liver disorders. BDL is a well-known laboratory model that mimics the complex mechanisms of liver cirrhosis. The in vitro model of bile duct ligation in rodents has been used as an experimental method in research for many years. The first protocol for this model was proposed more than three decades ago in which cannulation, occlusion, and ligation cause cirrhosis with changes in liver tissue of rodents that became similar to those seen in human cirrhosis [23]. This animal model causes apoptosis and necrosis of liver cells and ultimately liver fibrosis and cirrhosis through bile cytotoxic components such as bile lipophilic acid and also responsible for plasma membrane damage and oxidative stress. In this study, we evaluated the protective and antioxidant effects of OM in BDL rats and analyzed the role of this plant in expressing the liver genome in these rats.

The serum levels of AST, ALT, and ALP are important indexes for the clinical manifestation of cholestasis/cirrhosis. As expected, according to Tahan et al. study [24], the level of biochemical markers including AST, ALT, ALP, and total bilirubin increased significantly in the BDL-V group compared to the SC-V group. AST and ALT mainly distributed in the hepatocytes; they release into the blood circulation when hepatocytes membrane are damaged by bile duct obstruction; then these changes in liver enzymes cause loss of liver tissue integrity and cell death [25, 26]. In our study, OM treatment slightly decreased the elevated level of AST and ALT induced by BDL. The results suggested that OM extract, due to its antioxidant properties, slightly reduces liver damage by inhibiting the formation of free radicals, thus preventing the release of liver enzymes into the blood.

One of the standard indicators for assessing collagen formation in liver tissue is the measurement of hydroxyproline levels. In a study by Zhao et al., hydroxyproline level in the BDL-V group was higher than the SC-V group [27]. Our findings also showed a significant increase in hydroxyproline level in the BDL-V group compared to SC-V. In the present study, the hydroxyproline content in the liver of BDL rats receiving OM extract was significantly lower than the BDL-V group (*P* < 0.05). Accumulation of bile acids damages hepatocytes; then, inflammatory cells produce inflammatory cytokines and ROS, leading to inflammatory responses and collagen deposition. Presumably, OM hydroalcoholic extract reduces collagen production by trapping ROS, thereby reducing the hydroxyproline content in the liver tissue.

An imbalance between oxidants and the antioxidant system in cells causes oxidative stress. Oxidative stress and related events are the most important mechanisms associated with cholestasis/cirrhosis. BDL not only increased free radicals but also decreased antioxidant activity [28]. High level of MDA indicates an increase in lipid peroxidation, which ultimately causes tissue damage as well as the over-formation of free radicals by impairing the antioxidant defense mechanism [29, 30]. According to the study of Zhao et al., MDA levels increased in both serum and liver tissue in the BDL group [27]. Fang et al. indicated a significant increase in MDA level in BDL-induced cholestasis rats compared with the SC group [31]. The present data showed that the plasma MDA of cholestatic rats significantly increased compared to the SC-V group, and OM had no effect on plasma MDA levels.

FRAP represents the antioxidative capacity of plasma [20]. In the present study, the plasma FRAP level of the BDL-V group had a significant increase compared to SC-V group. In 2020, Ghaeni et al. found that OM extract significantly increased FRAP in the high-fat diet induced hepatosteatosis [32]. FRAP mostly supports the antioxidant activity of OM plant, which is mostly due to its phenolic compounds [33]. Our findings showed that FRAP levels in the BDL + OM group slightly increased compared to BDL-V animals. This difference may be due to the lower dose of OM used in our study.

Total thiols contain endogenous antioxidant compounds that also have radical scavenging effects and metal chelators, which, for this reason, has played an important role against oxidative stress [34]. According to a study by Sadeghi et al. in 2019, our results showed that the level of TSH in the BDL-V group significantly decreased as compared to the SC-V group [20], whereas administration of OM had no significant effect on the level of TSH compared to the BDL-V group.

It has been reported that the administration of medicinal plants and natural compounds with antioxidant activity such as hydroalcoholic extract of Watercress [20], *Ziziphus mauritiana* leaf [35], polydatin [31], quercetin [36], and Marjoram oil [37] can protect the liver against oxidative damage caused by cholestasis. SOD and CAT are antioxidants that prevent the production of free radicals and also work as part of the first line of defense in the living cells [30]. Zhao et al. showed that SOD activity was significantly increased in the OM treated groups compared with the control group [27]. Other studies have reported that *Origanum vulgare* consumption significantly increases the activity of the antioxidant enzymes such as SOD and CAT [33, 38]. Our results showed that the SOD activity did not change in all groups but CAT activity significantly increased in the BDL + OM group as compared to only BDL-V rats. CAT is enriched in liver tissue which is responsible for catalyzing the decomposition of hydrogen peroxide to water and oxygen. It seems that phenolic compounds stimulate CAT activity which reduces the number of free radicals, suggesting that OM has the antioxidant effects on the cholestatic liver.

One of the important elements in the onset and progression of liver fibrosis is inflammation and immune responses. Inflammatory cells are a significant source of cytokines that can initially mediate inflammatory responses and later cause tissue fibrosis [39]. TNF-*α* and TGF-*β* as proinflammatory cytokines signal the expression of several inflammatory mediators and the regulation of the inflammatory response, which are increased by oxidative stress [40, 41]. Recent findings showed that hepatic TNF-*α* and TGF-*β* genes expression was increased in liver injury [36, 42], which is consistent with our results. In our work, OM extract reduced hepatic TGF-*β* gene expression, which was increased by BDL-V. However, TNF-*α* expression was not significantly different from BDL-V. TGF-*β* is a profibrotic cytokine that increases the inhibition of metalloproteinases, thereby reducing the degradation of the extracellular matrix and increasing collagen synthesis. Accordingly, OM extract may suppress the inflammatory response in rats by inhibiting the proinflammatory marker (TGF-*β*) and/or controlling the oxidant status.

## 5. Conclusion

The data of the present study showed that oral treatment with OM (300 mg/kg) produced a moderate hepatoprotective and inflammatory activity in the cholestatic rats. In fact, to confirm this finding, further studies with different doses of extracts are needed.

## Data Availability

The data supporting the findings of this study are available within the article.
